# Extramedullary Hematopoiesis in a Sentinel Lymph Node as an Early Sign of Chronic Myelomonocytic Leukemia

**DOI:** 10.1155/2015/594970

**Published:** 2015-04-16

**Authors:** Joslin M. Bowen, Anamarija M. Perry, Erin Quist, Mojtaba Akhtari

**Affiliations:** ^1^Physicians Laboratory, 4840 F Street, Omaha, NE 68117, USA; ^2^Department of Pathology, University of Manitoba, MS559S Thorlakson Building, 820 Sherbrook Street, Winnipeg, MB, Canada R3A 1R9; ^3^Department of Pathology and Microbiology, University of Nebraska Medical Center, 985900 Nebraska Medical Center, Omaha, NE 68198, USA; ^4^Jane Anne Nohl Division of Hematology and Center for the Study of Blood Diseases, University of Southern California (USC) and Norris Cancer Center, USC University Hospital, 1441 Eastlake Avenue, Norris Topping Tower 3463, MC 9172, Los Angeles, CA 90033-9172, USA

## Abstract

Chronic myelomonocytic leukemia (CMML) is a clonal hematopoietic malignancy with features of both a myeloproliferative neoplasm and a myelodysplastic syndrome. Even though extramedullary leukemic infiltration is common in CMML patients, lymph node involvement has rarely been reported in the literature. We present an unusual case of a 72-year-old female who was found to have extramedullary hematopoiesis (EMH) in a sentinel lymph node that was excised during mastectomy for lobular breast carcinoma. One year later bone marrow biopsy was performed due to persistent anemia, thrombocytopenia, and monocytosis and the patient was diagnosed with CMML. Our case illustrates the importance of recognizing EMH in a lymph node during routine histological examination, especially in adults. Proliferation of bone marrow elements in a lymph node, in a patient with no known hematologic disorder, should trigger immediate bone marrow evaluation, as this could be the first clue in diagnosing underlying bone marrow disorder.

## 1. Introduction

Chronic myelomonocytic leukemia (CMML) is a clonal hematopoietic malignancy characterized by overlapping features of both a myeloproliferative neoplasm and a myelodysplastic syndrome [1, 2]. The 2008 World Health Organization (WHO) classification defines CMML using the following criteria: (1) persistent monocytosis of >1 × 10^9^ in the peripheral blood (PB), (2) absence of Philadelphia chromosome and* BCR-ABL1* fusion gene, (3) no rearrangement of* PDGFRA* or* PDGFRB* in the cases with eosinophilia, (4) fewer than 20% blasts and/or promonocytes in the PB and bone marrow (BM), and (5) dysplasia involving one or more myeloid lineages [1]. Clinically, PB and BM are always involved. Extramedullary leukemic infiltration can be seen in CMML, and it is most commonly encountered in spleen and liver [1, 3]. Disease less frequently involves other sites such as skin and genitourinary tract [4–6]. Lymph node enlargement in CMML has been reported in the older literature (i.e., before the advent of 2001 WHO classification [7]) in 14% to 30% of patients [3, 8]; however, there are very few reports in the literature that histologically describe involved lymph nodes [8–11].

Herein, we report an unusual case of a 72-year-old female who was found to have extramedullary hematopoiesis in a sentinel lymph node, excised for lobular carcinoma of the breast, and was diagnosed with CMML one year later. Extramedullary hematopoiesis in a sentinel lymph node can be considered as an early sign of CMML.

## 2. Case Presentation

**Figure 1:**
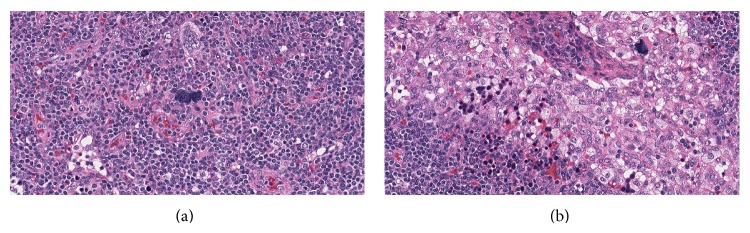
(a) Paracortical area of the lymph node with extramedullary hematopoiesis (H&E ×200); (b) extramedullary hematopoiesis in the lymph node sinus with prominent red blood cell precursors and megakaryocytes (H&E ×200).

**Figure 2:**
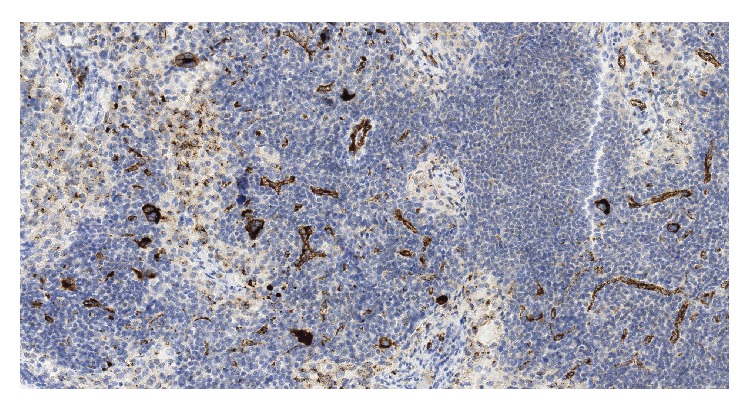
Immunohistochemical stain for Factor VII shows positivity in megakaryocytes (×100).

**Figure 3:**
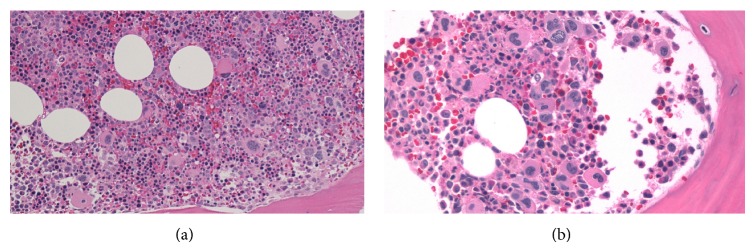
(a) Hypercellular bone marrow with increase in all three hematopoietic lineages (H&E ×100); (b) prominent dysplasia and clustering of megakaryocytes (H&E ×200).

A 72-year-old lady was found to have a 1 cm spiculated lesion on screening mammography. She underwent mastectomy and sentinel lymph node excision for a biopsy proven lobular breast carcinoma. Sentinel lymph node showed overall preserved architecture with follicular and paracortical hyperplasia and dilated sinuses. In the paracortical area (Figure 1(a)) and the sinuses (Figure 1(b)) there were small clusters of immature erythroid cells, as well as scattered megakaryocytes, consistent with extramedullary hematopoiesis (EMH). Megakaryocytes showed dyspoietic changes including hyperlobation and hyperchromatic nuclei. There was no significant dyspoiesis in erythroid precursors. Myeloid precursors were not prominent component of the EMH. There was no morphological increase in blasts or promonocytes. Immunohistochemical stain for Factor VIII further highlighted the megakaryocytes (Figure 2). One year later, the patient was referred to a hematologist for worsening anemia (hemoglobin 10.5 g/dL; normal range, 11.5–15.5 g/dL) and thrombocytopenia (platelets ranged from 89,000 to 124,000/*μ*L; normal range, 150,000–400,000/*μ*L), and persistent monocytosis (absolute monocyte count 1.2 × 10^3^/*μ*L; normal range, 0.1–1.0 × 10^3^/*μ*L). Of note, absolute monocyte count at the time of the sentinel lymph node biopsy one year prior was 1.5 × 10^3^/*μ*L. The patient underwent a bone marrow biopsy which showed markedly hypercellular marrow for patient's age (80% cellularity; Figure 3(a)). Dyspoiesis was present in all three hematopoietic lineages but most prominent in megakaryocytes that showed clustering with numerous small and hypolobated forms (Figure 3(b)). There was prominent monocytosis with 18% monocytes by differential count. Blasts (including promonocytes) were not significantly increased and by differential counts were <5% in the PB and <10% in the BM. There was moderate (grade 2/3) diffuse reticulin fibrosis. Conventional cytogenetic studies showed the following karyotype: 46,XX,del(20)(q11.2q13.3)[20]. Fluorescence in situ hybridization studies showed del20q12 and del20q13. Molecular studies were negative for* JAK2* V617F mutation and for* BCL-ABL1* fusion gene. The patient was diagnosed with CMML-1. She became symptomatic due to progressive anemia and was initially treated with 6 cycles of azacitidine, to which she responded; however, she lost her response after 8 cycles. Her regimen was then switched to decitabine, to which she had a fair response. Her general condition started deteriorating; she had prolonged neutropenia and passed away due to septic shock.

## 3. Discussion

Extramedullary hematopoiesis is a result of conditions that disrupt the bone marrow microenvironment, facilitating the egress of precursor cell, with an increase in circulating mature and immature marrow elements. After birth, there is normally very little proliferation of hematopoietic elements outside the marrow. Extramedullary hematopoiesis occurs in a number of conditions, including benign hematologic disorders (e.g., thalassemia, hereditary spherocytosis, sickle cell anemia, and congenital dyserythroblastic anemia), hematopoietic neoplasms, stromal disorders of the marrow (e.g., osteopetrosis), nonhematopoietic tumors, infectious, and storage diseases, as well as disorders of the circulation [12, 13]. In a case of hematopoietic neoplasm that involves extramedullary site, better term to use is neoplastic myeloid proliferation (NMP), to differentiate it from EMH that occurs as a consequence of benign disorders. Neoplastic myeloid proliferation can be seen in association with myeloproliferative neoplasms, myelodysplastic syndromes, myelodysplastic/myeloproliferative neoplasms, and other myeloid derived malignancies [13]. Extramedullary hematopoiesis has been reported in almost all body sites, including lymph nodes [12–14].

In the case of our patient, EMH was an incidental finding in a sentinel lymph node that was excised due to lobular carcinoma of the breast. Proliferation of bone marrow elements in a lymph node is not considered normal in an adult and should prompt an immediate search for an underlying bone marrow disorder [13]. However, our patient was not evaluated for hematologic neoplasm until a year later when she was referred to a hematologist for worsening cytopenias and was diagnosed with CMML. Median survival of CMML patients is 20–40 months and multiple drugs are being used in treatment of this disease [1, 2]. Delay in diagnosis and treatment could potentially shorten patient's survival. In children, the presence of EMH in lymph nodes could indicate undiagnosed benign hematologic disorder, and lymphadenopathy with EMH may be an initial manifestation [13]. Even though lymph node involvement, detected by imaging studies, is not uncommon in patients with myeloproliferative/myelodysplastic neoplasms, routine lymph node biopsies are not performed in these patients [3, 8, 14]. Sudden appearance of lymphadenopathy can also indicate transformation to acute myeloid leukemia/myeloid sarcoma [11].

Several authors have reported EMH in axillary lymph nodes following neoadjuvant therapy for breast cancer [15–17]. This finding is likely related to use of bone marrow suppressing chemotherapeutic agents along with granulocyte colony stimulating factors in these patients [16]. Extramedullary hematopoiesis in a lymph node is a potential diagnostic pitfall in these cases as it could be mistaken for metastatic carcinoma. Furthermore, presence of EMH can be especially problematic when evaluating lymph nodes during frozen section where morphology is frequently less than optimal [15, 16]. Our patient did not receive neoadjuvant chemotherapy prior to lymph node sampling, but it is important to keep this “phenomenon” in differential diagnosis in patients with a history of chemotherapy.

In conclusion, our case illustrates the importance of recognizing EMH in a lymph node during routine histological examination, especially in adults, since it can be an early sign of an underlying hematological malignancy. Proliferation of bone marrow elements in a lymph node, in a patient with no known hematologic disorder, should trigger immediate bone marrow evaluation, as this could be the first clue in diagnosing underlying bone marrow disorder.
